# Experiments Showing the Effects of Morphine and Its Acetate upon Persons in Health

**Published:** 1829-08

**Authors:** 

**Affiliations:** Turin.


### MORPHINE.

*Experiments showing the Effects of Morphine and its Acetate upon Persons in Health.* By Dr. Beraudi, of Turin. (*Annali Univ. di Med.*)

Among the numerous experiments which have been lately made for the purpose of determining the therapeutic or the injurious effects arising from morphine and the acetate of morphine, but few trials have been made upon persons in health, to show with precision the phenomena which would be produced by the exclusive influence of this preparation of opium. In order to elucidate this important question, Dr. Beraudi, and MM. Rebuni, Crispo, and Allinio, tried a series of experiments upon themselves. M. Manfredi prepared the acetate of morphine with much care, and assured himself that the salt obtained was not a subacetate.

*First series of Experiments.*

The above gentlemen assembled after dinner.

1. At three o'clock precisely, M. Allinio, ætat. twentytwo, of a bilious temperament, pulse sixty-six, swallowed one eighth of a grain of the acetate of morphine in two ounces of distilled water. He had scarcely swallowed the liquid, when he had a bitter and rather acrid taste in the fauces. In five minutes, great pain in the epigastric region, disposition to sleep, with difficult respiration. In thirty minutes, an abundant sweat broke out over the whole body, pupils much dilated, pulse ninety-four. Thirty-three minutes, drowsiness and pain in the forehead. Fifty minutes, lips livid, face flushed, conjunctivæ turgid, darting pain in the forehead. Fifty-two minutes, pain in the region of the bladder; stupidity of countenance, but the eyes very brilliant; excessive thirst, and great feeling of lassitude in the legs. At a quarter-past four o'clock, M. A. felt itching of the skin, constant pain in the genito-urinary organs, and particularly in the right spermatic cord; heaviness of the forehead. These symptoms continued till near seven o'clock: at that time there were great pain in the epigastrium, nausea, and inclination to vomit. He did not sleep till half-past two in the morning, and during the interval he had been much agitated, and tormented by acute pain in the head and in the umbilical region. From that hour till half-past six A.M. he slept profoundly. He then awoke with a dull sensation in the forehead, and shortly after he had evacuations from the bowels.

2d. M. Crispo, æt. twenty-one, of a sanguine and bilious temperament, pulse sixty, also took, at three o'clock P.M., in two ounces of distilled water, one sixth of a grain of the acetate of morphine. Immediately after, he had an excessively bitter taste. In four minutes, nausea. In twenty minutes, stupor, dilated pupils, borborygmus, pulse seventy-nine. Fifty minutes, cheeks of a circumscribed red colour, stupid appearance, eyes brilliant, cold sweat over the body, heaviness of the head, and disposition to sleep. Four o'clock: nausea, followed by drowsiness, terminated by a more abundant and general sweat. Half-past five: pain in the bladder, slight diarrhœa, pain in the epigastrium. Tranquil sleep throughout the night.

3. M. Rebuni, æt. nineteen, of a sanguine temperament and robust constitution, took, at three P.M., one eighth of a grain of acetate of morphine, in two ounces of distilled water. Pulse sixty-three. With the exception of an acceleration of the pulse, which was raised to 108 in half an hour, and a slight redness of the edges and tip of the tongue, this young man experienced no particular effects.

4. At three P.M., Dr. Beraudi swallowed, in two ounces of distilled water, half a grain of the acetate of morphine. Before the experiment his pulse was sixty-five. Immediately he felt a very bitter taste, followed by a painful sensation at the epigastrium, which extended to the bladder. In five minutes, an abundant and general sweat. Fifteen minutes, nausea, difficult breathing, heaviness, pale tongue, pulse sixty-six and undulating. Thirty minutes: pupils much dilated, pain in the occiput, with weight over the eyes, conjunctivæ injected. Thirty-five minutes: insupportable pain on the right side of the head, restlessness, face flushed, (in health the countenance was pale;) sweat dropped from the face; stupid and downcast air, itching of the skin. Four o'clock: violent headach, face almost livid; in a few minutes, agitated and frequently interrupted sleep for three hours. On awaking, great pain at the epigastrium and bladder, urine limpid, and voided in very small quantities, although there was great desire to make water. Dr. Beraudi got up, and, upon walking, felt continued nausea. The pain in the epigastrium soon ceased, and was felt in the umbilical region; some food was then taken, after which the pain in the epigastrium immediately returned with greater severity. Ten in the evening: diarrhœa, with shooting pains in the region of the stomach, umbilicus, and bladder, by which all repose was banished for the night; skin very dry, and the itching insupportable. The diarrhœa continued all the following morning, and ceased, with the other symptoms, at noon.

The uneasiness experienced by Dr. Beraudi and M. Allinio prevented them from continuing their experiments until the next day.

*Second series of Experiments.*

On the 10th of September, at eight o'clock, the experiments were continued; each was made fasting.

1st. At the above time, M. Allinio swallowed a quarter of a grain of acetate of morphine, in an ounce of distilled water. Pulse sixty-six. He experienced a bitter taste, and in twenty minutes the end of the tongue was extremely red, and the pupils dilated. In about half an hour, acute pain in the forehead, burning heat of the skin, pulse eighty, extreme lassitude of the limbs. About nine o'clock, face animated, lips livid, excessive thirst, some efforts to vomit, which soon disappeared. After this nothing remarkable occurred, and the various symptoms gradually disappeared.

2d. At eight o'clock M. Rebuni also took a quarter of a grain of acetate of morphine. The liquid appeared to him excessively bitter. Tongue became rather red, but no other particular phenomenon was presented; while his colleagues experienced a general weariness all day.

3d. At the same hour M. Crispo took a third of a grain of acetate of morphine, in two ounces of distilled water. Pulse sixty-five. Immediately after, a strong bitter taste and slight pain in the epigastrium; in five minutes, a burning sensation in the back of the throat; and, in fifteen minutes later, a decided redness of the end and sides of the tongue, with dilatation of the pupils. Twenty-five minutes, extreme weariness of the limbs, the back, the neck, and shortly after of all the joints; pulse sixty-eight, and very irregular; conjunctivæ injected, face much flushed, lips pale; no disturbance in the cerebral functions, and the ordinary state of health was soon restored. During the night, however, the dilatation of the pupils continued, even when he looked directly at the sun. The next morning, there was an eruption on the surface of the body.

4th. At eight in the morning, Dr. Beraudi, in his turn, took two thirds of a grain of the acetate of morphine, in two ounces of distilled water. The liquid was very bitter. Immediately after, pain in the epigastrium and on the right side of the head; pupils very much dilated, although opposed to the sun; pulse rose from sixty-one to eighty-six; nausea, efforts at vomiting, extreme weariness in the joints, conjunctivæ injected, lips pale, eyes sparkling, dull pain in the frontal region, particularly the right. Ten o'clock: heavy sleep, cheeks flushed, end and sides of tongue red, violet coloured in the centre; at the same time a painful sensation, difficult to describe, was felt in the region of the stomach, the umbilicus, and bladder, with a feverish pulse. At eleven o'clock, some greenish matter was vomited; Dr. Beraudi then got up, and walked about. All the symptoms disappeared in the course of the day.

It is worthy of remark, that each of these individuals, the next morning, felt a sensation of pain and obstruction in the back part of the throat, without any unusual redness being perceived in that region.

*Third series of Experiments.*

On the 11th of October, at half-past eight in the morning, Messrs. Allinio, Crispo, Rebuni, Sella, and Dr. Beraudi, recommenced their experiments, fasting.

1st. M. Allinio then took a grain of the acetate of morphine, in a sufficient quantity of distilled water: a painful sensation in the epigastrium was almost immediately felt, and in a moment dilatation of the pupils; the eyes appeared, as it were, leaping from the orbits; face red, lips pale, the point of the tongue red, the roof of the palate of a scarlet red, and rather painful. Nine o'clock: violent headach; pulse, at first sixty-eight, was then seventy-eight. Pain in the epigastrium increasing progressively, with a strong sensation of heaviness in the frontal region, followed by disturbed sleep; face moist with sweat, pulse eighty-eight. At eleven o'clock, awoke; pain in the forehead, epigastrium, and bladder, but, above all, a strong feeling of general fatigue and dull pains in the joints. This state remained till dinner-time, when M. Allinio ate with some little appetite, but nausea soon came on, and a desire to vomit. He had a good night, and on the morrow was attacked with slight diarrhœa. The next morning, about half-past eight, violent pain was felt in the front of the right side, with a cold and copious sweat along the back; two fainting fits; pupils excessively dilated; tongue pale, mouth clammy and bitter. The latter symptoms disappeared during the day, after two very painful evacuations.

It should be observed, that the constipation remained the whole of the day on which the acetate of morphine was taken.

2d. The same day, at half-past eight in the morning, M. Crispo swallowed half a grain of the acetate of morphine, in a little lukewarm alcohol. Hitherto the pulse had been at eighty-four. A very bitter taste was soon experienced; in a quarter of an hour, efforts at vomiting; in half an hour, extreme dilatation of the pupils: the pulse then ninety-four. Ten o'clock: face flushed, sensation of an appetite, which was soon appeased. Dinner-time: M. Crispo again ate a little. Contrary to custom, he immediately experienced a strong desire to sleep, and slumbered until five o'clock: awoke with headach in the forehead, acute and shooting pain in the umbilical region, which lasted till night. The next morning, in health, but the face and a great part of the body was covered with pimples.

3d. M. Rebuni, fasting, took, at half-past eight, half a grain of morphine, in a little alcohol. Pulse sixty-six. In half an hour, pupils dilated, pulse eighty-two; at ten o'clock, pulse ninety. With the exception of some slight pains in the epigastrium and abdomen, no symptoms arose worthy of notice.

4th. M. Sella, who had hitherto been a mere observer, wished in his turn to make an experiment upon himself. At nine o'clock, he took half a grain of morphine: he had breakfasted half an hour. He immediately felt a very bitter taste, and the face became much flushed. Half-past nine, pulse sixty-four, appetite, with great pain in the epigastrium, followed by pain in the umbilical region. Soon after, a very painful sensation in the loins; and, towards four o'clock, hiccough, which lasted three quarters of an hour; after which, extreme weakness, followed by heat and excessive itching over the whole body. Health was soon restored.

5th. On the same day and hour, Dr. Beraudi took a grain of morphine, in a sufficient quantity of alcohol. The liquid had a very bitter taste. The pulse, before the experiment, sixty, in less than half an hour rose to eighty. Dr. B. took a little chocolate. Ten o'clock: pain in the epigastrium, face flushed, eyes bloodshot, tongue rather red, headach, followed by calm sleep, which lasted till eleven o'clock. Nausea then came on, with slight vomiting. If, by the red tint it produces, nitric acid will discover morphine, it is certain there was morphine in what was vomited. This sickness did not prevent Dr. Beraudi taking food at dinner-time, but, soon after a light meal, headach, with intense pain in the epigastrium, obliged him to go to bed. Profound sleep until half-past eight in the evening. During the night, disturbed sleep, with fever, headach, continual itching of the skin, very copious diarrhœa, which continued next day. For three days, pain in the stomach was almost constantly felt.

The decided uneasiness produced by the above-mentioned doses prevented the experimenters from taking the morphine and its acetate in larger doses.

These experiments do not support the opinion of M. Bally, according to whom morphine produces no effect upon either the mouth, pharynx, or tongue. They confirm the experiments made by M. Chevallier upon himself.*

